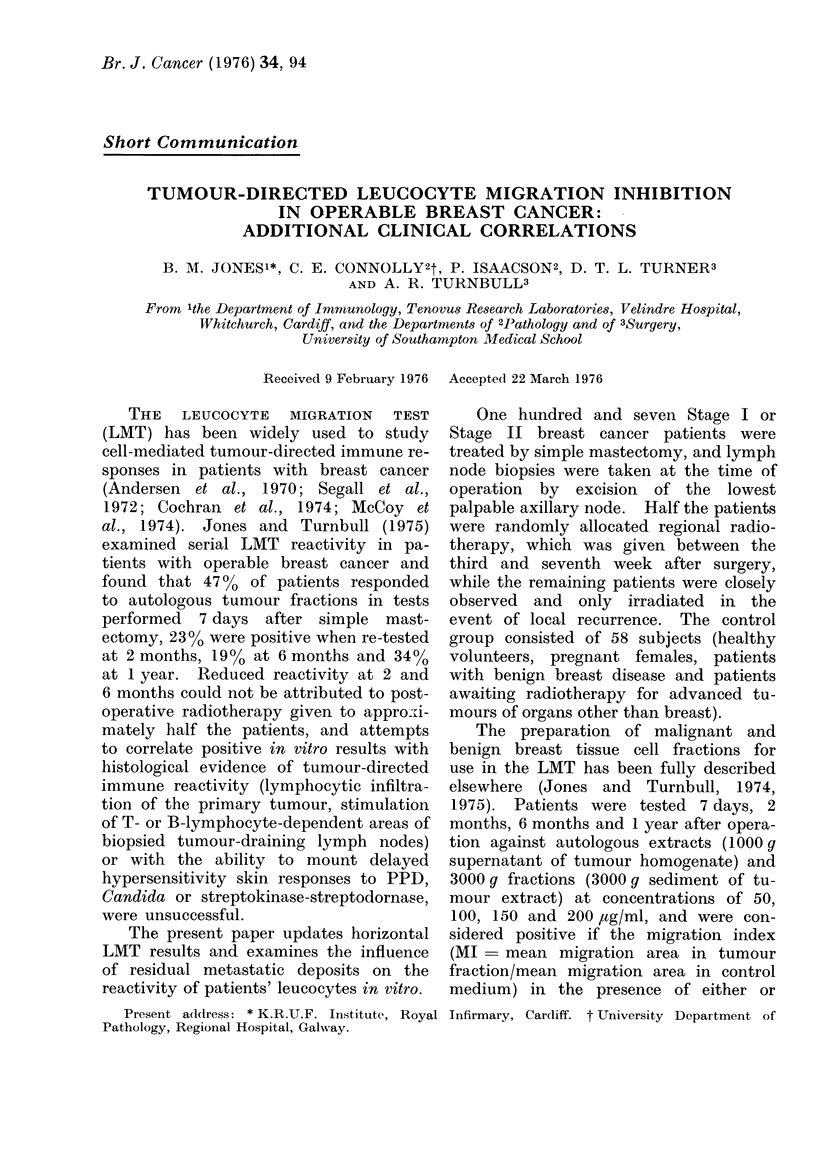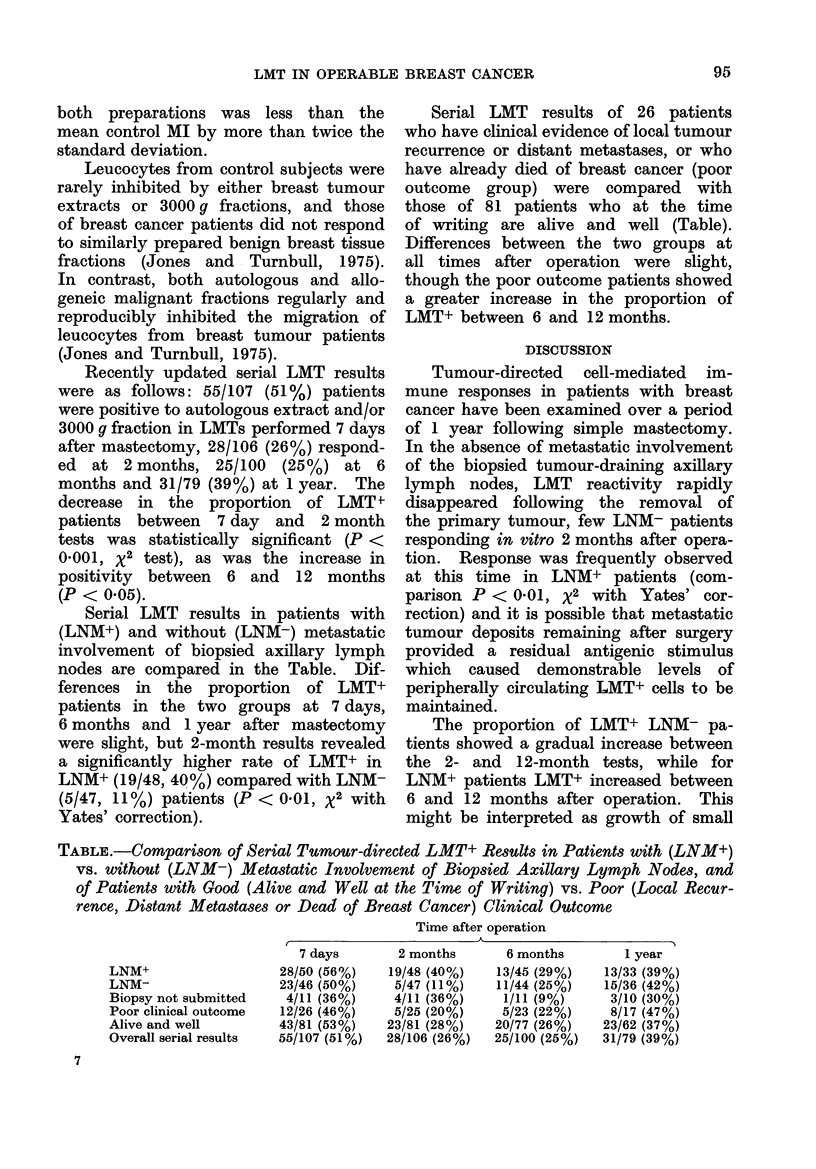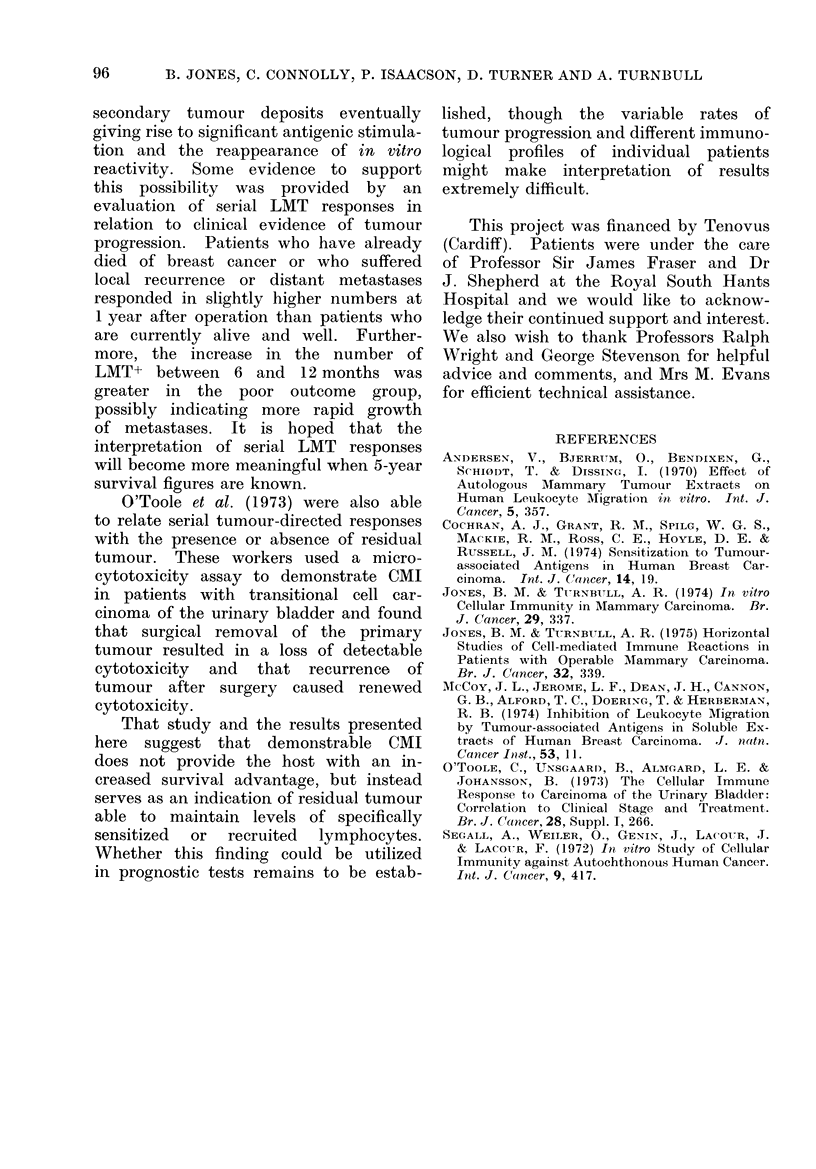# Tumour-directed leucocyte migration inhibition in operable breast cancer: additional clinical correlations.

**DOI:** 10.1038/bjc.1976.128

**Published:** 1976-07

**Authors:** B. M. Jones, C. E. Connolly, P. Isaacson, D. T. Turner, A. R. Turnbull


*Br. J. Cancer* (1976) **34**, 94

### *Short Communication*

### TUMOUR-DIRECTED LEUCOCYTE MIGRATION INHIBITION IN OPERABLE BREAST CANCER: ADDITIONAL CLINICAL CORRELATIONS

B. M. JONES[1]*, C. E. CONNOLLY[2]†, P. ISAACSON[2], D. T. L. TURNER[3] AND A. R. TURNBULL[3]

*From [1]the Department of Immunology, Tenovus Research Laboratories, Velindre Hospital, Whitchurch, Cardiff, and the Departments of [2]Pathology and of [3]Surgery, University of Southampton Medical School*

Received 9 February 1976 Accepted 22 March 1976

THE LEUCOCYTE MIGRATION TEST (LMT) has been widely used to study cell-mediated tumour-directed immune responses in patients with breast cancer (Andersen *et al.*, 1970; Segall *et al.*, 1972; Cochran *et al.*, 1974; McCoy *et al.*, 1974). Jones and Turnbull (1975) examined serial LMT reactivity in patients with operable breast cancer and found that 47% of patients responded to autologous tumour fractions in tests performed 7 days after simple mastectomy, 23% were positive when re-tested at 2 months, 19% at 6 months and 34% at 1 year. Reduced reactivity at 2 and 6 months could not be attributed to post-operative radiotherapy given to approximately half the patients, and attempts to correlate positive *in vitro* results with histological evidence of tumour-directed immune reactivity (lymphocytic infiltration of the primary tumour, stimulation of T- or B-lymphocyte-dependent areas of biopsied tumour-draining lymph nodes) or with the ability to mount delayed hypersensitivity skin responses to PPD, *Candida* or streptokinase-streptodornase, were unsuccessful.

The present paper updates horizontal LMT results and examines the influence of residual metastatic deposits on the reactivity of patients' leucocytes *in vitro*.

One hundred and seven Stage I or Stage II breast cancer patients were treated by simple mastectomy, and lymph node biopsies were taken at the time of operation by excision of the lowest palpable axillary node. Half the patients were randomly allocated regional radiotherapy, which was given between the third and seventh week after surgery, while the remaining patients were closely observed and only irradiated in the event of local recurrence. The control group consisted of 58 subjects (healthy volunteers, pregnant females, patients with benign breast disease and patients awaiting radiotherapy for advanced tumours of organs other than breast).

The preparation of malignant and benign breast tissue cell fractions for use in the LMT has been fully described elsewhere (Jones and Turnbull, 1974, 1975). Patients were tested 7 days, 2 months, 6 months and 1 year after operation against autologous extracts (1000 *g* supernatant of tumour homogenate) and 3000 *g* fractions (3000 *g* sediment of tumour extract) at concentrations of 50, 100, 150 and 200 μg/ml, and were considered positive if the migration index (MI = mean migration area in tumour fraction/mean migration area in control medium) in the presence of either or

Present address: * K.R.U.F. Institute, Royal Infirmary, Cardiff. † University Department of Pathology, Regional Hospital, Galway.

both preparations was less than the mean control MI by more than twice the standard deviation.

Leucocytes from control subjects were rarely inhibited by either breast tumour extracts or 3000 *g* fractions, and those of breast cancer patients did not respond to similarly prepared benign breast tissue fractions (Jones and Turnbull, 1975). In contrast, both autologous and allogeneic malignant fractions regularly and reproducibly inhibited the migration of leucocytes from breast tumour patients (Jones and Turnbull, 1975).

Recently updated serial LMT results were as follows: 55/107 (51%) patients were positive to autologous extract and/or 3000 *g* fraction in LMTs performed 7 days after mastectomy, 28/106 (26%) responded at 2 months, 25/100 (25%) at 6 months and 31/79 (39%) at 1 year. The decrease in the proportion of LMT$^+$ patients between 7 day and 2 month tests was statistically significant ($P < 0{\cdot}001$, $\chi^2$ test), as was the increase in positivity between 6 and 12 months ($P < 0{\cdot}05$).

Serial LMT results in patients with (LNM$^+$) and without (LNM$^-$) metastatic involvement of biopsied axillary lymph nodes are compared in the Table. Differences in the proportion of LMT$^+$ patients in the two groups at 7 days, 6 months and 1 year after mastectomy were slight, but 2-month results revealed a significantly higher rate of LMT$^+$ in LNM$^+$ (19/48, 40%) compared with LNM$^-$ (5/47, 11%) patients ($P < 0{\cdot}01$, $\chi^2$ with Yates' correction).

Serial LMT results of 26 patients who have clinical evidence of local tumour recurrence or distant metastases, or who have already died of breast cancer (poor outcome group) were compared with those of 81 patients who at the time of writing are alive and well (Table). Differences between the two groups at all times after operation were slight, though the poor outcome patients showed a greater increase in the proportion of LMT$^+$ between 6 and 12 months.

### DISCUSSION

Tumour-directed cell-mediated immune responses in patients with breast cancer have been examined over a period of 1 year following simple mastectomy. In the absence of metastatic involvement of the biopsied tumour-draining axillary lymph nodes, LMT reactivity rapidly disappeared following the removal of the primary tumour, few LNM$^-$ patients responding *in vitro* 2 months after operation. Response was frequently observed at this time in LNM$^+$ patients (comparison $P < 0{\cdot}01$, $\chi^2$ with Yates' correction) and it is possible that metastatic tumour deposits remaining after surgery provided a residual antigenic stimulus which caused demonstrable levels of peripherally circulating LMT$^+$ cells to be maintained.

The proportion of LMT$^+$ LNM$^-$ patients showed a gradual increase between the 2- and 12-month tests, while for LNM$^+$ patients LMT$^+$ increased between 6 and 12 months after operation. This might be interpreted as growth of small

TABLE.—*Comparison of Serial Tumour-directed LMT$^+$ Results in Patients with (LNM$^+$) vs. without (LNM$^-$) Metastatic Involvement of Biopsied Axillary Lymph Nodes, and of Patients with Good (Alive and Well at the Time of Writing) vs. Poor (Local Recurrence, Distant Metastases or Dead of Breast Cancer) Clinical Outcome*

| | Time after operation | | | |
|---|---|---|---|---|
| | 7 days | 2 months | 6 months | 1 year |
| LNM$^+$ | 28/50 (56%) | 19/48 (40%) | 13/45 (29%) | 13/33 (39%) |
| LNM$^-$ | 23/46 (50%) | 5/47 (11%) | 11/44 (25%) | 15/36 (42%) |
| Biopsy not submitted | 4/11 (36%) | 4/11 (36%) | 1/11 (9%) | 3/10 (30%) |
| Poor clinical outcome | 12/26 (46%) | 5/25 (20%) | 5/23 (22%) | 8/17 (47%) |
| Alive and well | 43/81 (53%) | 23/81 (28%) | 20/77 (26%) | 23/62 (37%) |
| Overall serial results | 55/107 (51%) | 28/106 (26%) | 25/100 (25%) | 31/79 (39%) |

secondary tumour deposits eventually giving rise to significant antigenic stimulation and the reappearance of *in vitro* reactivity. Some evidence to support this possibility was provided by an evaluation of serial LMT responses in relation to clinical evidence of tumour progression. Patients who have already died of breast cancer or who suffered local recurrence or distant metastases responded in slightly higher numbers at 1 year after operation than patients who are currently alive and well. Furthermore, the increase in the number of $LMT^+$ between 6 and 12 months was greater in the poor outcome group, possibly indicating more rapid growth of metastases. It is hoped that the interpretation of serial LMT responses will become more meaningful when 5-year survival figures are known.

O'Toole *et al.* (1973) were also able to relate serial tumour-directed responses with the presence or absence of residual tumour. These workers used a microcytotoxicity assay to demonstrate CMI in patients with transitional cell carcinoma of the urinary bladder and found that surgical removal of the primary tumour resulted in a loss of detectable cytotoxicity and that recurrence of tumour after surgery caused renewed cytotoxicity.

That study and the results presented here suggest that demonstrable CMI does not provide the host with an increased survival advantage, but instead serves as an indication of residual tumour able to maintain levels of specifically sensitized or recruited lymphocytes. Whether this finding could be utilized in prognostic tests remains to be established, though the variable rates of tumour progression and different immunological profiles of individual patients might make interpretation of results extremely difficult.

This project was financed by Tenovus (Cardiff). Patients were under the care of Professor Sir James Fraser and Dr J. Shepherd at the Royal South Hants Hospital and we would like to acknowledge their continued support and interest. We also wish to thank Professors Ralph Wright and George Stevenson for helpful advice and comments, and Mrs M. Evans for efficient technical assistance.

### REFERENCES

Andersen, V., Bjerrum, O., Bendixen, G., Schiødt, T. & Dissing, I. (1970) Effect of Autologous Mammary Tumour Extracts on Human Leukocyte Migration *in vitro*. *Int. J. Cancer*, **5**, 357.

Cochran, A. J., Grant, R. M., Spilg, W. G. S., Mackie, R. M., Ross, C. E., Hoyle, D. E. & Russell, J. M. (1974) Sensitization to Tumour-associated Antigens in Human Breast Carcinoma. *Int. J. Cancer*, **14**, 19.

Jones, B. M. & Turnbull, A. R. (1974) *In vitro* Cellular Immunity in Mammary Carcinoma. *Br. J. Cancer*, **29**, 337.

Jones, B. M. & Turnbull, A. R. (1975) Horizontal Studies of Cell-mediated Immune Reactions in Patients with Operable Mammary Carcinoma. *Br. J. Cancer*, **32**, 339.

McCoy, J. L., Jerome, L. F., Dean, J. H., Cannon, G. B., Alford, T. C., Doering, T. & Herberman, R. B. (1974) Inhibition of Leukocyte Migration by Tumour-associated Antigens in Soluble Extracts of Human Breast Carcinoma. *J. natn. Cancer Inst.*, **53**, 11.

O'Toole, C., Unsgaard, B., Almgard, L. E. & Johansson, B. (1973) The Cellular Immune Response to Carcinoma of the Urinary Bladder: Correlation to Clinical Stage and Treatment. *Br. J. Cancer*, **28**, Suppl. I, 266.

Segall, A., Weiler, O., Genin, J., Lacour, J. & Lacour, F. (1972) *In vitro* Study of Cellular Immunity against Autochthonous Human Cancer. *Int. J. Cancer*, **9**, 417.